# Kidney biopsy in west of Iran: Complications and histopathological findings

**DOI:** 10.4103/0971-4065.53325

**Published:** 2009-04

**Authors:** M. Rahbar

**Affiliations:** Department of Pathology, Emam Reza Hospital, Kermanshah, Iran

**Keywords:** Glomerulonephritis, renal biopsy, renal parenchymal disease

## Abstract

In this retrospective study, we reviewed the medical records and histopathology findings of 135 patients who underwent renal biopsies at two special hospitals affiliated to Kermanshah medical university during a six-year period (2003-2007). All were performed using Tru-Cut needle under ultrasound guidance. Twenty four specimens were unsatisfactory. There were 38 males (34.2%) and 73 females (65.7%) in 111 patients with adequate specimens (each specimen has more than 5 glomeruli); the mean age was 16.5 years (range 2-64 years). Side effects of the renal biopsies included pain at the site of biopsy in 2 (2.7%), gross hematuria in 1 (0.9%). Nephrotic syndrome was the most common indication for biopsy followed by acute renal failure of unknown etiology and nephritic syndrome. Primary glomerular disease was reported in 78 patients (70.2%) and also secondary glomerular disease in 33 patients (29.7%). Among the primary glomerulonephritis disease, minimal change disease and membranous glomerulonephritis were the commonest findings in children below the age of 16 years. Minimal change disease ranked first in adults whole membranous glomerular disease and focal segmental glomerulosclerosis were more common in the elderly. In all patients lupus glomerular disease was the commonest secondary glomerular disease. We conclude that study on renal biopsy makes final diagnosis which is associated with an acceptably low rate of complications in our practice, and in all, the patterns of renal histology in our study vary slightly from those reported from other countries.

## Introduction

Epidomiologic studies on renal biopsies is the best way to monitor glomerular disease trends and to make policies for early detection and to control of existing glomerular disease. Therefore histological examination of the biopsied kidneys have remained the gold standard for renal diagnosis yet.

In this study we report our experience with renal biopsy and the histopathological patterns of renal disease in a referral hospitals in west of Iran over six years period.

This retrospective study aimed at answering three questions: What are the indications of renal biopsy in the West of Iran? What is the rate of unsatisfactory renal needle biopsy specimen? Do we have a different complication rate? And does the pattern of renal disease differ from other countries?

## Materials and Methods

A retrospective study of all patients who underwent percutaneous renal biopsies in our center over the last six years was performed. We reviewed all renal biopsies and referral slides pertaining to renal parenchymal disease in the archives of the department of pathology in two hospitals by two pathologists and one nephropathologist from 2003 to 2007.

The indications for performing the biopsies were nephrotic syndrome, nephritic syndrome, renal failure of unknown etiology, persistent or recurrent asymptomatic hematuria or proteinuria.

Patients were excluded if they had bleeding problems, uncontrolled hypertension, single kidney, and morbid obesity or were unco-operative. Prior to the biopsy procedure, clotting profile including platelet count, bleeding time, clotting time, prothrombin and partial thromboplastin time were performed. Only a small numbers of patients needed correction for one or more abnormalities of these parameters before performing the biopsies.

Nephrologists using Tru-cut^®^ needles performed all biopsies guided by ultra-sonographic localization of the kidneys. Two specimens were subjected to only light and immunoflorescent microscopic studies (for studing of immunoglobulines, complement contentes and fibrins deposits). Each specimen consists of atleast two fragments and before staining each specimen was evaluated for enough numbers of giomeruli by light microscopic examination. Each fragment should be consisted about more than five numbers glomeruli. Electron microscopy was not available for use in this study. Biopsy samples were considered satisfactory for diagnosis if they contained five or more glomeruli. We examined with light microscopy stained sections; one with Hematoxylin and Eosin, one with periodic Schiff, one with Massons Trichrome and one with Jones Silver.

The most common complications of the procedure were pain at biopsy site in 2 patients, microscopic hematuria in eight numbers patients, gross hematuria in one patient, and hematuria requiring blood transfusion in zero patient. None of patients needed open surgical intervention or nephrectomy and there is no death during the procedure of renal biopsy.

## Results

During the past six years, a total number of 111 adequate renal biopsies were performed in our centers. thirty one (27.9%) were less than 16 years old (group I), 56 (50.4%) were more than 16 years old and less than 50 years old (Group II) and 24 (21.6%) more than 50 years old (group III) in two patters; primary and secondary glomerulonephritis. There were 38 males (34.2%) and 73 females (65.7%). The male to female ratio was 0.5.

Indication for performing the biopsies among different age groups were nephrotic syndrome in 47 patients (42.3%), renal failure (unknown etiology) in 36 patients (32.4%), nephritic syndrome in 20 patients (18.0%), asymptomatic hematuria in 8 (7.2%) [Table 1].

**Table 1:** Indication of renal biopsy

Indication of renal biopsy	N	%
Nephrotic syndrome	47	42.3
Acute renal failure (unknown etiology)	36	32.4
Nephritic syndrome	20	18.0
Asymptomatic hematuria	8	7.2
Total	111	100

As expected, minimal change disease(MCD) and membranous glomerulonephritis (MGN) were the commonest patterns in groups I and II. Membranous glomerulonephritis and focal segmental glomerulonephritis (FSGN) were more common in group III [Tables 2 and 3]. The most common complications of the procedure were pain at the biopsy site in two (1.8%) patients, gross hematuria in one (0.9%), hematuria requiring blood transfusion in zero (0%) patient. None of the patients needed open surgical intervention or nephrectomy and there were no deaths due to the procedure [Table 4].

**Table 2:** Prevalence of different types of primary GN in three age groups

Primary GN	Less than 16 yrs		Between 16-50 yrs		More than 50 yrs	
			
	N	%	N	%	N	%
MCD	12	48	20	50	2	9.5
MGN	7	28	9	22.5	10	47.6
FSGS	2	8	3	7.5	3	14.2
MPGN	2	8	1	2.5	2	9.5
IgA nephropathy	2	8	1	2.5	0	0
RPGN	0	0	1	2.5	1	4.7
Total	25(100%)					

**Table 3:** Prevalence of different types of secondary GN in three age groups

Secondary GN	Less than 16 yrs		Between 16-50 yrs		More than 50 yrs	
			
	N	%	N	%	N	%
Lopus nephritic	4	66.6	10	62.5	1	33.3
Diabetic nephropathy	0	0	6	37.5	2	66.6
PIGN	2	33.3	0	0	0	0
Total	N = 6 (100%)		% = 29.7			

**Table 4:** Clinical significant complications after needle renal biopsy

Complications	N	%
Local pain	2	1.8
Gross hematuria	1	0.9
Others	0	0
Total	3	2.7

## Discussion

In our study the nephrotic syndrome was the commonest indication of performing biopsy (42.3% of cases) which is higher than other studies.[1–3] The overall complication rate in this study was 2.7%. local pain at the biopsy site was in 1.8% and gross hematuria was in 0.9%. These rates are slightly lower than other studies. In one study, the overall complication rate was 7%, the rate of pain was 4% and that of gross hematuria was 3%.[4] The patterns of glomerulopathy in our study differs among different age groups. Minimal change disease (MCD) was encountered in 48% of children and young adults which is less than 76.5% reported by some studies[5,6] but higher than others.[3,7–9] The relatively low incidence of MCD among nephrotic children in our study can be explained by the fact that patients in this group were given empirical courses of steroids first, biopsy was done only for those who did not respond showed poor response or had frequent relapses.

Minimal change disease was the most common histological pattern encountered in the adult group(groug II) followed by membranous glomerulonephritis; both accounted for 50% and 22.5%, respectively. This is in contrast to lower incidence of these two entities in the international and regional reports,[1,2,5,10,12–18] however the findings are similar to other studies.[19,20] Nephrotic patients in the elderly groups (group III) has different histopathology. Membranous glomerulonephritis (MGN) and focal segmental glomrulosclerosis(FSGN) accounted for the majority of cases 47.6%,14.2% respectively. Other researchers have reported membranous nephropathy and minimal as chang disease are common patterns in these age groups.[18] IgA nephropathy was diagnosed in three (2.7%) patients of all groups (8% in group I and 2.5% in group II and 0% in group III). Al Menawy *et al.*, reported that 2.7% of children < 16 years had IgA nephropathy[7] and in another study on 300 cases that conducted by Abdurrahman reported a 3% incidence of IgA nephropathy.[21] We conclude that renal biopsy was with minimal rate of complications in our practice, the patterns of renal disease differ among different age groups and vary slightly from other countries and lopus nephritis is frequent in adult groups (group II). Diabetic nephritis is common in group III. Nevertheless, Rapid progressive glomeruonephritis is reported in children and young adults (group I).
